# Polymorphisms in hormone metabolism and growth factor genes and mammographic density in Norwegian postmenopausal hormone therapy users and non-users

**DOI:** 10.1186/bcr3337

**Published:** 2012-10-27

**Authors:** Merete Ellingjord-Dale, Eunjung Lee, Elisabeth Couto, Ali Ozhand, Samera Azeem Qureshi, Solveig Hofvind, David J Van Den Berg, Lars A Akslen, Tom Grotmol, Giske Ursin

**Affiliations:** 1 Department of Nutrition, University of Oslo, P.O. Box 1046, Blindern, 0316 Oslo, Norway; 2 Department of Preventive Medicine, Keck School of Medicine, University of Southern California, 1441 Eastlake Avenue, Los Angeles, CA 90089, USA; 3 Cancer Registry of Norway, P.O. Box 5313, Majorstuen, 0304 Oslo, Norway; 4 Department of Medical Epidemiology and Biostatistics, Karolinska Institutet, P.O. Box 281, SE-17177 Stockholm, Sweden; 5 Oslo and Akershus University College of Applied Sciences, P.O. Box 4, St. Olavs plass, 0130 Oslo, Norway; 6 The Gade Institute, Section for Pathology, University of Bergen, P.O. Box 7804, NO-5020 Bergen, Norway

## Abstract

**Introduction:**

Mammographic density (MD) is one of the strongest known breast cancer risk factors. Estrogen and progestin therapy (EPT) has been associated with increases in MD. Dense breast tissue is characterized by increased stromal tissue and (to a lesser degree) increased numbers of breast epithelial cells. It is possible that genetic factors modify the association between EPT and MD, and that certain genetic variants are particularly important in determining MD in hormone users. We evaluated the association between MD and 340 tagging single nucleotide polymorphisms (SNPs) from about 30 candidate genes in hormone metabolism/growth factor pathways among women who participated in the Norwegian Breast Cancer Screening Program (NBCSP) in 2004.

**Methods:**

We assessed MD on 2,036 postmenopausal women aged 50 to 69 years using a computer-assisted method (Madena, University of Southern California) in a cross-sectional study. We used linear regression to determine the association between each SNP and MD, adjusting for potential confounders. The postmenopausal women were stratified into HT users (EPT and estrogen-only) and non-users (never HT).

**Results:**

For current EPT users, there was an association between a variant in the *prolactin *gene (*PRL*; rs10946545) and MD (dominant model, Bonferroni-adjusted *P *(Pb) = 0.0144). This association remained statistically significant among current users of norethisterone acetate (NETA)-based EPT, a regimen common in Nordic countries. Among current estrogen-only users (ET), there was an association between rs4670813 in the *cytochrome P450 *gene (*CYP1B1*) and MD (dominant model, Pb = 0.0396). In never HT users, rs769177 in the *tumor necrosis factor (TNF) *gene and rs1968752 in the region of the *sulfotransferase *gene (*SULT1A1/SULT1A2)*, were significantly associated with MD (Pb = 0.0202; Pb = 0.0349).

**Conclusions:**

We found some evidence that variants in the *PRL *gene were associated with MD in current EPT and NETA users. In never HT users, variants in the *TNF *and *SULT1A1/SULT1A2 *genes were significantly associated with MD. These findings may suggest that several genes in the hormone metabolism and growth factor pathways are implicated in determining MD.

## Introduction

Mammographic density (MD) is the radiodense or white area on a mammogram. MD reflects the amount of fibrous stroma and epithelium in the breast [1,2]. MD has been associated with stromal growth factors [3], as well as the number of epithelial cells, but not with increased epithelial cell proliferation [4].

MD is one of the strongest known breast cancer risk factors [5]. Women with MD of 75% or greater have a risk of breast cancer that is four to five times higher than women of the same age with little or no density (≤ 5%) [6].

Several studies have suggested that MD is at least partially inherited. Studies of twins suggest that genetic factors explain 30 to 60% of the variation in MD [7-9]. A number of studies have assessed the role of genetic variants identified through genome-wide association studies (GWAS) of breast cancer on MD [10,11], but the results of these studies are inconsistent. A recent meta-analysis of GWAS on MD identified one variant (rs10995190) in the *zinc finger protein 365 *gene (*ZNF365*) [12], but this variant only explained about 0.5% of the variance in MD. Thus, a large proportion of the variation is yet to be explained.

MD decreases with older age and menopause [13], and increases in postmenopausal women randomized to hormone treatment [14]. Parity [15] and early age at first full-term pregnancy are both associated with reduced MD [16]. Because of the clear role of hormones on MD, it has been suggested that genes involved in pathways that regulate steroid hormone synthesis and metabolism, hormone receptors, or genes in growth factor pathways may play a role. However, genetic association studies of candidate pathways either involving hormones or growth factors, have been few so far [17,18]. Many studies have focused on selected variants, and these have not yielded consistent results [19-28]. It seems likely that such variants could determine how women metabolize hormone therapy (HT), or determine the downstream effect of hormones, and therefore some variants would only be important in women taking HT. Few of the studies so far have examined this association according to HT status [18,21].

Therefore, we decided to investigate which genetic variants were the most important in explaining MD in HT users, and whether the association between genetic variants and MD was modified statistically by HT use. We evaluated the association between MD and 340 single nucleotide polymorphisms (SNPs) from about 30 putative genes in hormone metabolism and growth factor pathways.

## Materials and methods

### Participants

Characteristics of the study participants and details about the study have previously been described [29-31]. Women selected for the study had attended the Norwegian Breast Cancer Screening Program (NBCSP) at least once. This national screening program invites all women aged 50 to 69 years to undergo a mammographic examination every two years. The attendance rate is 76.2% [32].

A questionnaire on various breast cancer risk factors, enclosed within the NBCSP invitation, was sent to a random sample of 17,050 women living in the counties of Oslo, Akershus and Hordaland in 2004. Menstrual and reproductive history, use of oral contraceptive and menopausal hormonal therapy, family history of breast cancer, current weight and height were assessed by the standardized questionnaire. A total of 12,056 (71%) of the invited women attended the screening program and 7,941 (66%) returned a completed questionnaire.

A subset (7,174) of the 7,941 women who had completed the health questionnaire was asked to complete a food frequency questionnaire and to provide us with two buccal swabs. Of these, 3,484 women (49%) returned the dietary questionnaire and 3,728 returned buccal swabs. We requested mammograms from the various radiological facilities and we focused the requests on mammograms from women who had completed a food frequency questionnaire and had had a screen film mammography in 2004. About 300 women from Oslo had undergone digital mammography. These women were not included in the current study as assessments from digital images tend to yield somewhat low percent MD compared with digitized screen film mammograms.

We obtained information on HT use and analog screening mammograms from the year 2004 on 2,876 women. Of these, 124 women were excluded for the following reasons; 17 women had had a previous cancer (12 = breast, 5 = ovarian), the breast area could not be determined on mammograms from 3 women, 28 had incomplete data on age, and 73 incomplete data on body mass index (BMI) (height = 46/weight = 67). Three women were excluded because they used progesterone-only HT. After the exclusions, a total of 2,752 women with mammograms and HT data were left for analysis. All the participants signed an informed consent and the study was approved by the Regional Ethics Committee and the Norwegian Data Inspectorate.

### Mammographic density analysis

Mammograms (left craniocaudal) were scanned using a high-resolution Kodak Lumisys 85 scanner with automatic feeder (Kodak, Rochester, NY, USA). Computer-assisted readings of absolute areas of dense and non-dense tissues, as well as percent density, were performed using the Madena software [33]. This method provides a continuous measure of density and a separate estimate of the absolute areas of dense and non-dense tissues. Only analog mammograms were included due to concern that the digitally obtained images would not yield comparable density readings. The density assessments were performed by an experienced reader (G.U.), whereas a research assistant trained by G.U. conducted the breast area measurements. Both readers were blinded to all subject characteristics. Percent MD was calculated by dividing the absolute breast density by the total breast area and multiplying it by 100. Some suggest that absolute density is the optimal method [34], but the majority of the studies on MD have used percent density [6], and we therefore limited these results to percent MD.

### DNA collection and extraction

Three thousand seven hundred and twenty-eight (3,728) women returned buccal swabs out of which 3,317 were genotyped. Of these, 241 women had to be excluded due to a sample call rate < 80%, yielding 3,076 women. Of these, we had risk factor information and mammograms collected in 2004 on 2,397 women. DNA was extracted from buccal swabs using standard modified protocol for the QIAamp DNA blood kit (Qiagen, Valencia, CA, USA).

### Selection of SNPs

For *AR*, *COMT*, *CYP1A1/CYP1A2*, *CYB1B1*, *ESR2*, *HSD17B1*, *IGFBP1/IGFBP3*, *IL6*, *PGR*, *PPARG*, *PRL*, *SULT1A1/SULT1A2*, *SULT1E1*, *TGFB1*, *TNF *and *VEGF*, we selected tag SNPs to capture the genetic variation in each gene. We selected linkage disequilibrium tagging SNPs across each gene, from 20 kb upstream of 5' untranslated region (UTR) to 10 kb downstream of the 3' UTR. Because this study shared an analysis platform with a study of Chinese women, HapMap Caucasians of European descent (CEU) data (release 24) [35] and HapMap Han Chinese in Beijing (CHB) data (release 24), as well as the Snagger [36] or modified Tagger approach were used to capture all common SNPs (minor allele frequency ≥ 5%) in Caucasians or Chinese with minimum pair wise r^2 ^> 0.8. For, *ADH1C*, *AKR1C4*, *CSHL1*, *CYP17A1*, *CYP19A1*, *ESR1*, *FGFR2*, *GHRHR*, *HCV3289988*, *HSD3B1/HSD3B2*, *POU5F1*, *PRLR*, *SHBG *and *SRD5A2*, we selected one or a few SNP(s) of interest for each gene (Table 2).

**Table 2:** List of genes on single nucleotide polymorphisms (SNPs) genotyped and tagging SNP approach.

Genes	Number of SNPs successfully genotyped for each gene	Tagging SNP approach
ADH1C	1	No
AKR1C4	1	No
CSHL1	1	No
CYP17A1	1	No
CYP19A1	2	No
ESR1	12	No
FGFR2	1	No
GHRHR	1	No
HCV3289988	1	No
HSD3B1/HSD3B2	3	No
POU5F1	1	No
PRLR	3	No
SHBG	13	No
SRD5A2	1	No

AR	6	Yes
COMT	29	Yes
CYP1A1/CYP1A2	13	Yes
CYP1B1	17	Yes
ESR2	29	Yes
HSD17B1	3	Yes
IGFBP1/IGFBP3	29	Yes
IL6	19	Yes
PGR	32	Yes
PPARG	32	Yes
PRL	34	Yes
SULT1A1/SULT1A2	5	Yes
SULT1E1	1	Yes
TGFB1	11	Yes
TNF	15	Yes
VEGF	21	Yes

*See methods for details on how tagging SNPs were selected.

### Genotyping of SNPs

Genotyping was done using an Illumina BeadLab System (Illumina Inc., San Diego, CA, USA) and GoldenGate™ Genotyping technology in the University of Southern California Genomics Center. Samples were run in a 96-well format using Illumina Sentrix Array technology on a BeadArray Reader. BeadStudio Software (version 3.0.9) with Genotyping Module (version 3.0.27) (Illumina) was used for analyzing scanned samples. The SNPs with < 85% call rates were excluded: this resulted in the exclusion of 4% of SNPs. The genotyping concordance rate based on 41 duplicate samples was 84%. Out of 340 SNPs in the hormone metabolism and growth factor pathways, 18 SNPs departed from Hardy-Weinberg equilibrium (HWE) (*P *< 0.001), leaving 322 SNPs for further analysis. None of these excluded 18 SNPs were associated with MD regardless of the hormone use status (data not shown).

### Menopausal status and HT use

The classification of postmenopausal women was limited by our questionnaire where women were asked if they had undergone a) complete cessation of menstruation of at least six months b) previous bilateral oophorectomy c) hysterectomy without bilateral oophorectomy d) used HT before menopause. Out of the 2,397 women, we excluded 342 pre- and perimenopausal women. We also excluded 19 women who had undergone simple hysterectomy without bilateral oophorectomy since these women could not be classified on menopausal status. Our final sample size was 2,036 postmenopausal women. Excluding women with menopause within the past year (*N *= 177) yielded essentially unchanged results, and did not alter the order of the most important SNPs (results not shown). We therefore present analyses using the six month definition of menopause.

HT use was assessed by asking two questions about 1) ever use of HT with a proposed list of HT preparations and 2) current use of HT. If a woman had used the specified HT for more than three months at the time of completing the questionnaire, she was considered a current user. A woman could have used both estrogen-only (ET) and combined estrogen and progestin therapies (EPT) in her lifetime, but only one of these currently. Current EPT users were subdivided further into norethisterone acetate (NETA) regimen users or non-NETA users.

### Statistical analyses

We used multivariate linear regression to examine the relation between percentage MD and SNPs. The MD variable was treated in a continuous manner, without any transformations, as the model's residuals satisfied the normality and homoscedasticity assumptions. We adjusted all analyses for age at screening (continuous) and BMI (kg/m^2^, continuous).

We ran these analyses on both additive and dominant models. However, because the results were largely similar between the two genetic models, and few women were homozygote for the variants of many SNPs, we only present the dominant model (results available upon request). We conducted analyses, with and without Bonferroni adjustments for multiple comparisons (the number of SNPs per gene) within each stratum defined by hormone use. We also ran a test for heterogeneity between HT users and non-users and present the *P *values for this interaction (*P*_int_). In the results section, we specifically comment on results where the stratum-specific two-sided *P *values are less than 0.01 and the Bonferroni-adjusted *P *values (Pb) are less than 0.05.

We estimated least squares means (marginal means) on MD and all our explanatory variables among never, current and past EPT users. Least squares means are the group mean after having controlled for a covariate (age and BMI). We subdivided HT users into ET or EPT. EPT users were further subdivided into NETA users or non-users.

## Results

Characteristics of the study population by strata of EPT use (no, past and current) are shown in Table 1. As previously described, MD was higher in current EPT users than in past or never users [31]. There were statistically significant differences in percent density, BMI and in age at screening between never, past and current EPT users. Table 2 shows the number of SNPs successfully genotyped for each gene we investigated.

**Table 1:** Characteristics of no, past and current estrogen and progestin therapy (EPT) users (*N *= 2,036).

	No EPT (*N *= 1,176)			Past EPT (*N *= 612)			Current EPT (*N *= 248)			
			
	N	Mean^1^	SE^2^	N	Mean^1^	SE^2^	N	Mean^1^	SE^2^	P^3^
**Mammographic density (%)**	1,176	17.07	0.46	612	18.99	0.45	248	23.34	0.51	0.0001
**Age at screening (years)**	1,176	58.19	0.18	612	59.22	0.15	248	57.33	0.15	0.0001
**Body mass index (kg/m^2^)**	1,176	25.44	0.13	612	24.91	0.10	248	24.15	0.11	0.0001
**Age at menarche (years)**	1,169	13.16	0.04	607	13.36	0.04	246	13.31	0.04	0.1025
**Age at first pregnancy (years)**	1,049	21.75	0.25	540	22.3	0.24	225	22.28	0.24	0.3602
**Number of children**	1,114	2	0.03	587	2	0.03	239	2	0.03	0.7252
**Education (years)**	1,164	12.75	0.10	605	13.01	0.10	245	12.85	0.10	0.2807

^1^Adjusted for age and BMI, ^2^standard error, ^3^analysis of variance.

Among all postmenopausal women, there was no association between SNPs and MD after Bonferroni adjustments (Table S1 in Additional file 1). Among postmenopausal never HT users, there was an association between MD and a variant in the *tumor necrosis factor *(*TNF*) gene (rs769177; Pb = 0.020), and with a variant in the *sulfotransferase *(*SULT1A1/SULT1A2*) gene (rs1968752; Pb = 0.035) (Table S2 in Additional file 2).

**Figure 1:**
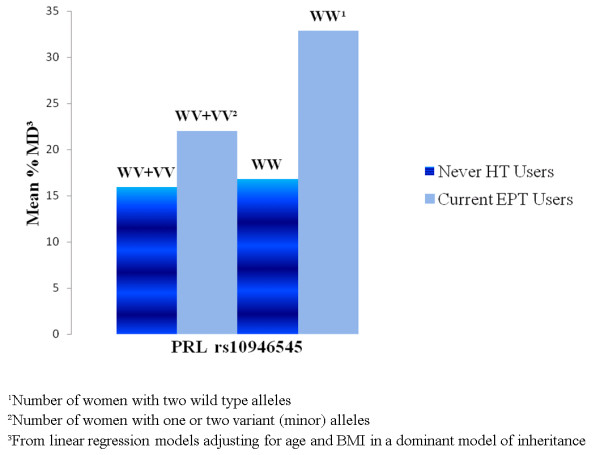
**Mean percent mammographic density by *prolactin *(*PRL*) rs10946545 genotype and hormone therapy use**. A figure that presents mean percent mammographic density by the *PRL *gene (rs10946545) in women with two wild-type alleles and with one or two variant (minor) alleles in never hormone therapy (HT) users and in current estrogen and progestin therapy (EPT) users.

For postmenopausal current ET users (Table 3) there was an association between a variant in the *cytochrome P450 *(*CYP1B1*) gene (rs4670813, Pb = 0.0396) and MD. In current EPT users (Table 4) a variant in the *prolactin *(*PRL*) gene (rs10946545, Pb *= *0.0144) was significantly associated with MD. The results from the analysis of all SNPs in all women combined, and in ET users and EPT users separately, as well as the interaction tests are shown in Table S3 in Additional file 3. Mean percent mammographic density was highest in homozygous wild-type carriers of *PRL *rs10946545 who currently used EPT (Figure 1).

**Table 3:** Top twenty single nucleotide polymorphism (SNPs) in postmenopausal current estrogen-only users (*N *= 78) that showed the strongest heterogeneity between never hormone therapy users and estrogen-only users.

Gene	SNP	SNPs per gene	Alleles	WW	WV+VV	Beta^1^	SE	P^1^	Pb^2^	Pint^3^
PPARG	rs1175543	32	A/G	42	36	-9.80	3.00	0.0017	0.0535	0.0028
CYP1B1	rs4670813	17	G/A	25	53	-9.99	3.17	0.0023	**0.0396**	0.0059
CYP1B1	rs162550	17	G/C	44	34	8.19	3.11	0.0103	0.1753	0.0068
ESR2	rs1256063	29	G/A	65	13	12.27	4.00	0.0030	0.0868	0.0069
IGFBP1/IGFBP3	rs1496497	29	G/A	47	31	-8.08	3.13	0.0119	0.3464	0.0155
IL6	rs6949149	19	G/T	70	8	-12.82	4.97	0.0119	0.2256	0.0206
TGFB1	rs8179181	11	A/T	48	30	7.91	3.16	0.0144	0.1579	0.0214
ESR1	rs2295190	12	G/T	51	27	-6.45	3.36	0.0590	0.7086	0.0293
PPARG	rs1151996	32	A/C	36	42	-7.18	3.11	0.0236	0.7554	0.0520
CYP1B1	rs1625	17	T/G	49	29	6.74	3.25	0.0418	0.7098	0.0557
TGFB1	rs1982072	11	A/T	35	43	-6.32	3.17	0.0498	0.5478	0.0577
CYP1A1/CYP1A2	rs11072507	13	C/G	39	39	7.26	3.24	0.0281	0.3656	0.0578
CYP1B1	rs162557	17	G/A	47	31	6.05	3.21	0.0635	1.0791	0.0622
IGFBP1/IGFBP3	rs10241749	29	A/G	49	29	7.10	3.35	0.0375	1.0878	0.0657
PPARG	rs2120825	32	T/G	56	22	8.12	3.42	0.0201	0.6421	0.0733
COMT	rs12627876	29	C/T	77	1	26.61	13.83	0.0582	1.6881	0.0827
CYP1A1/CYP1A2	rs2606345	13	A/C	38	40	7.00	3.09	0.0263	0.3415	0.0886
IGFBP1/IGFBP3	rs4619	29	A/G	28	50	-6.51	3.31	0.0531	1.5400	0.0950
HSD3B1/HSD3B2	rs6428828	3	A/T	38	40	5.77	3.08	0.0650	0.1951	0.0954
PRL	rs1123886	34	T/C	36	42	6.22	3.17	0.0533	1.8109	0.2058

^1^From linear regression models using percent mammographic density as the outcome variable. Adjusted for age and BMI in a dominant model of inheritance. See Table S2 in Additional file 2 for the stratum-specific results in never HT users. ^2^Bonferroni-adjusted *P *values. ^3^*P *for interaction for ET vs. never HT users in all postmenopausal women (*N *= 2,036).

**Table 4:** Top twenty single nucleotide polymorphisms (SNPs) that showed the strongest heterogeneity in association between never hormone therapy users (*N *= 1,008) and postmenopausal current estrogen and progestin users (*N *= 248), by *P *for interaction.

Gene	SNP	SNPs per gene	Alleles	WW	WV+VV	Beta	SE	P^1^	Pb^2^	Pint^3^
PRL	rs10946545	34	G/A	219	29	10.86	3.04	0.0004	**0.0144**	0.0008
ESR2	rs12434245	29	C/T	216	32	-8.09	2.94	0.0063	0.1839	0.0034
TNF	rs3093553	15	T/G	228	20	-9.86	3.63	0.0070	0.1050	0.0046
CYP1A1/CYP1A2	rs3743	13	G/C	233	15	-9.79	4.16	0.0194	0.2524	0.0061
TNF	rs3093662	15	A/G	225	23	-8.53	3.42	0.0133	0.2002	0.0064
TNF	rs4947324	15	C/T	214	34	-8.53	2.89	0.0035	0.0520	0.0154
PRL	rs2744117	34	G/T	212	36	7.97	2.80	0.0048	0.1633	0.0206
ESR2	rs7159462	29	C/T	214	34	-6.61	2.87	0.0222	0.6447	0.0248
PRL	rs1156546	34	A/C	163	85	4.77	2.09	0.0230	0.7812	0.0249
SHBG	rs6258	13	C/T	246	2	23.25	11.09	0.0370	0.4815	0.0345
ESR2	rs10137185	29	C/T	209	39	-5.20	2.73	0.0575	1.6684	0.0425
PGR	rs569857	32	T/A	217	31	-5.69	3.02	0.0603	1.9306	0.0434
PPARG	rs4279078	32	G/A	203	45	6.09	2.58	0.0190	0.6082	0.0442
PPARG	rs2960420	32	C/G	111	137	-4.60	1.99	0.0219	0.6997	0.0458
VEGF	rs699946	21	A/G	159	89	-3.96	2.08	0.0587	1.2333	0.0719
PGR	rs7106686	32	G/A	188	60	-4.46	2.32	0.0559	1.7873	0.0854
PRL	rs9358533	34	C/T	207	41	6.45	2.66	0.0159	0.5413	0.0927
PGR	rs507141	32	G/A	185	63	4.40	2.28	0.0550	1.7594	0.1431
PGR	rs5181	32	C/G	208	40	-5.58	2.72	0.0411	1.3164	0.1753
PRL	rs1205960	34	C/T	140	108	-3.86	2.01	0.0559	1.8994	0.1765

^1^From linear regression models using percent mammographic density as the outcome variable. Adjusted for age and BMI in a dominant model of inheritance. ^2^Bonferroni-adjusted *P *values. ^3^*P *for interaction for EPT vs. never HT users in all postmenopausal women (*N *= 2,036).

NETA is an EPT-based regimen common in Nordic countries. The results remained statistically significant when analyses were restricted to NETA users (results not shown).

When we adjusted for parity (parous/nulliparous), the three *PRL *SNPs that were significant in the EPT users remained statistically significant before Bonferroni correction (rs10946545 *P *= 0.0028/rs2744117 *P *= 0.0218/rs1156546 *P *= 0.0345). None of these were statistically significant after Bonferroni adjustment. In the interaction test between these three SNPs and EPT, two of the three SNPs remained statistically significant (rs10946545 *P*_int _= 0.0051/rs1156446 *P*_int _= 0.0317) after adjustment for parity.

## Discussion

In this study of postmenopausal Norwegian women, we found that MD was associated with several variants in growth factor and hormone metabolism genes *(PRL*, *CYP1B1*, *SULT1A1*/*SULT1A2 *and *TNF*). However, different variants appeared to be important depending on the women's HT use.

There have been a number of studies of genetic variations and MD [18-28]. However, few studies have examined this association by HT use or tested the interaction with hormone use. Below, we discuss our results by subgroup of HT use.

Among postmenopausal never HT users (*N *= 1,008), we found associations between MD and variants in the *TNF *and the *SULT1A1/SULT1A2 *genes. *TNF *refers to a group of cytokines that are important in inflammation, as well as in growth, differentiation and cell death. *TNF *has been implicated in tumor regression [37,38], and there is some evidence that *TNF *genetic variants are associated with breast cancer risk [39]. Our findings are consistent with this. As far as we know, no other study has examined the association between *TNF *variants and MD. One cross-sectional study on postmenopausal American women found no statistically significant associations between circulating *TNF *levels and MD after adjustment for BMI [40]. However, the study did not take HT use into account. In our study, one *TNF *variant was important in never users and in all women, the latter association possibly because more than half of the women were never users. However, this variant was not important in EPT users, nor was the interaction between never users and EPT users statistically significant. Two other *TNF *variants showed statistical significance in the interaction between never users and EPT users, but not after Bonferroni adjustments. The exact role of *TNF *genetic variation on mammographic density is thus unclear, however, if inflammation is an important aspect of breast cancer development, then it seems reasonable that *TNF *variants may play a role.

Sulfotransferase 1A1 and 1A2 are enzymes that are encoded by the *SULT1A1 *and *SULT1A2 *genes. Sulfotransferase enzymes catalyze hormones, drugs and xenobiotic compounds [41]. In a review of 18 studies of SNPs located in genes of the estrogen pathway, *SULT1A1*/*SULT1A2 *did not show any association with MD on the overall study population [42]. In a study on American premenopausal women, it was concluded that SNPs in *SULT1A1 *locus may influence percent density [43]. In our study, *SULT1A1/SULT1A2 *was important in never users, but not in HT users. A possible explanation could be that the *SULT *variants interact with exposures important for MD in women not using hormones, although it may also be a chance finding.

There were few current ET users in our study (*N *= 78), but among them we observed a negative association between MD and rs4670813 (*CYP1B1*). *CYP1B1 *belongs to the cytochrome P450 super family of enzymes and is important in the metabolism of estrogen that may affect breast cancer risk. *CYP1B1 *catalyzes formation of potentially carcinogenic catechol estrogens and forms 4-hydroxylation of estrone [44]. *CYP1B1 *expression levels have also been reported to be lower in tumors than in adjacent benign tissue [45], suggesting that a key factor in breast carcinogenesis is not increased 4-hydroxylation but reduced estradiol metabolism. Thus, both increased and reduced *CYP1B1 *activity has been hypothesized to be associated with breast cell proliferation. In the SWAN study, *CYP1B1*; rs162555 was associated with a higher MD in pre- and early perimenopausal women [27]. In a cross-sectional study on postmenopausal women who participated in two randomized, double-blind, placebo-controlled studies, there was no evidence of an association between MD and genotypes in women randomized to ET [21]. In a case-control study of American postmenopausal women (PACE), the authors found an association between *CYP1B1*; rs1056827 and breast cancer risk in ever EPT users [46]. As long as the functional role of rs4670813 is unknown, the mechanism behind our observed negative association between MD and rs4670813 remains elusive.

The most significant findings in our study were the association between a variant in *PRL *gene (rs10946545) and MD in current EPT users (*N *= 248) and in users of NETA-based EPT. Prolactin, also known as luteotropic hormone, is a protein that in humans is encoded by the *PRL *gene [47]. It is produced by the pituitary gland and in lesser amounts by several other tissues, including breast tissue. Prolactin plays an important role in breast development, differentiation and lactation [48], but may also have procarcinogenic effects [49,50]. There are prolactin receptors in both normal breast tissue and in breast tumor tissue [51-53]. Our findings are consistent with the results in a case-control study on Polish women. In that study, two SNPs (rs7718468 and rs13436213) in the *prolactin receptor *(*PRLR*) gene were associated with breast cancer risk in postmenopausal women [54]. In our study, we found this association in current EPT users. Adjustments for parity did not obliterate the association, and the association was observed in strata of both parous and nulliparous women (results not shown). How parity and prolactin interact with hormones important for breast epithelial proliferation is not yet clear, but may be important in breast cancer development. Parity has previously been found to primarily reduce risk of estrogen receptor and progestin receptor positive (ER+PR+) cancer, while breastfeeding reduces risk of both ER+ PR+ and ER-PR- cancer [55]. Whether *PRL *plays a role in this interaction is unknown. When we adjusted for parity, the SNPs in the *PRL *gene remained statistically significant, suggesting that the number of children does not attenuate the association between variants in *PRL *and MD.

In a case-control study within the prospective Nurses' Health Study, plasma level of prolactin was positively associated with the risk of breast cancer [56]. Data from a randomized, placebo-controlled study suggested that EPT use in postmenopausal women increased the *PRL *concentration [57]. However, that study did not examine the role of genetic variations. In the first family linkage study on MD, a locus on chromosome 5p was associated with MD, and the *PRLR *is at a region surrounding this locus [28]. Taken together, current data suggest that both *PRL *and *PRLR *may be important for MD.

Summing up, our results indicate that a number of genetic variants that determine how women metabolize HT, or how HT exerts its effect on the breast, might be associated with MD in HT users.

### Strengths and weaknesses

There are several strengths of this study. First, our study investigated the association between genetic variations in the hormone metabolism and growth factor pathways and MD in HT users and non-users, and in ET and EPT users. Not many other studies have investigated these relations. Another strength of this study is the sample size (*N *= 2,036), and that it is population based. A third strength is that we had risk factor information available, although we only used age at screening, height/weight, hormone use and parity in this analysis. A weakness of the study is that we were not able to identify a study with similar types of hormone users, and thus have not been able to replicate our findings in another study. Further validation is therefore important. Another weakness is that we had only information on current height and weight (2004). We obtained the mammograms from three different screening facilities with different equipment and personnel. Even though the mammography procedures are standardized within the NBCSP, there is variation between radiographers on how to position and compress the breasts. Nevertheless, these potential sources of errors are not likely to be associated with genotype and would be expected to give a bias toward the null. We did not perform longitudinal studies, nor studies to establish the functional consequences of the SNPs, thereby restricting the ability to assess causation. To adjust for multiple comparisons we used Bonferroni adjustment. This is rather conservative, and we may thus have failed to identify other stratum-specific estimates that are biologically relevant.

## Conclusions

We found some evidence that variants in the *PRL *gene were associated with MD in current EPT and NETA users. Variants in *TNF *and *SULT1A1/SULT1A2 *were associated with MD in never HT users. These findings may suggest that several genes in the hormone metabolism/growth factor pathways are implicated in determining MD. Some genetic variants in combination with specific HT may increase MD in susceptible women. Exploring the functional role of the SNPs associated with MD will further clarify the biological mechanisms involved.

## Abbreviations

BMI: body mass index; CYP1B1: cytochrome P450 gene; EPT: estrogen and progestin therapy; ET: current estrogen-only users; GWAS: genome-wide association studies; HWE: Hardy-Weinberg equilibrium; HT: hormone therapy; MD: mammographic density; NBCSP: the Norwegian Breast Cancer Screening Program; NETA: norethisterone acetate; Pb: Bonferroni-adjusted *P*; PRL: prolactin gene; PRLR: prolactin receptor; SNP: single nucleotide polymorphism; SULT1A1/SULT1A2: sulfotransferase gene; TNF: tumor necrosis factor gene; UTR: untranslated region; ZNF365 gene: zinc finger protein 365.

## Competing interests

The authors declare that they have no competing interests.

## Authors' contributions

ME carried out the statistical analyses and drafted the manuscript. EL participated in the genetic data cleaning, analysis and interpretation of data and manuscript revision. EC provided expertise in the statistical program (SAS) and cleaned all the hormone therapy variables. AO took part in the genetic statistical analyses in SAS. DVDB coordinated tagging SNP selection and performed the genotyping. SQ participated in data cleaning and acquisition of data. LA, SH and TG contributed to the analysis and interpretation of data. GU participated in the design of the study and in SNP selection, and supervised the analysis and manuscript preparation. All authors have read and approved the final manuscript and revised it critically for important intellectual content.

## Supplementary Material

Additional file 1 **Table S1. Table S1 in Additional file 1. The frequency and the minor allele frequency (MAF) of the single nucleotide polymorphisms (SNPs) evaluated and association with percent mammographic density (Beta, se, *P*) for all postmenopausal women (*N *= 2,036)**. This is a list of all the SNPs and the frequency and minor allele frequency in the population for all postmenopausal women. Click here for file

Additional file 2 **Table S2. Table S2 in Additional file 2. Top twenty single nucleotide polymorphisms (SNPs) associated with percent mammographic density in postmenopausal never hormone therapy users (*N *= 1,008)**. This is a table of the top SNPs associated with MD in never HT users, sorted by *P *values. Click here for file

Additional file 3 **Table S3. Table S3 in Additional file 3. *P *values from analysis of all single nucleotide polymorphisms (SNPs) in all postmenopausal women combined, among estrogen therapy (ET) users only, and among combined estrogen and progestin therapy (EPT) users only, as well as results from interaction tests of ET users and EPT users versus never hormone therapy (HT) users**. A table that presents the *P *values for all women and the different strata in addition to *P *for interaction values between HT users and non-users. Click here for file
