# Supplementary material for: Polymorphisms in hormone metabolism and growth factor genes and mammographic density in Norwegian postmenopausal hormone therapy users and non-users
Source: Breast Cancer Res. 2012 Oct 27;14(5):R135. doi: 10.1186/bcr3337 (PMC4053113; doi:10.1186/bcr3337)
Supplement: Additional file 3 — Table S3. Table S3 in Additional file 3. P values from analysis of all single nucleotide polymorphisms (SNPs) in all postmenopausal women combined, among estrogen therapy (ET) users only, and among combined estrogen and progestin therapy (EPT) users only, as well as results from interaction tests of ET users and EPT users versus never hormone therapy (HT) users. A table that presents the P values for all women and the different strata in addition to P for interaction values between HT users and non-users. [file bcr3337-S3.DOC]

| **Additional file 3, Table S3. P-values from analysis of all SNPs in all postmenopausal women combined, among estrogen therapy (ET) users only, and among combined estrogen and progestin therapy (EPT) users only, as well as results from interaction tests of ET users and EPT users versus never hormone therapy (HT) users.** | | | | |  |  |  |
| --- | --- | --- | --- | --- | --- | --- | --- |
|  | | | |  |  |  |  |
|  |  |  |  |  |  |  |  |
| **Gene** | **SNP** | **P-values in all women¹** | **P-values in ET users¹** | **P-values in EPT users¹** | **P-values in Never users¹** | **P for the interaction ET vs. Never HT** | **P for the interaction EPT vs. Never HT** |
| PRL | rs10946545 | 0.1479 | 0.3120 | 0.0004 | 0.7378 | 0.4434 | 0.0008 |
| ESR2 | rs12434245 | 0.4233 | 0.1183 | 0.0063 | 0.4693 | 0.1792 | 0.0034 |
| TNF | rs3093553 | 0.3498 | 0.3196 | 0.0070 | 0.2162 | 0.2200 | 0.0046 |
| CYP1A1;CYP1A2 | rs3743 | 0.3483 | 0.3693 | 0.0194 | 0.4792 | 0.5264 | 0.0061 |
| TNF | rs3093662 | 0.3959 | 0.1459 | 0.0133 | 0.0837 | 0.0781 | 0.0064 |
| COMT | rs9332377 | 0.4795 | 0.5292 | 0.0969 | 0.0761 | 0.2822 | 0.0147 |
| TNF | rs4947324 | 0.9287 | 0.3734 | 0.0035 | 0.7142 | 0.3489 | 0.0154 |
| PPARG | rs12629751 | 0.9747 | 0.6779 | 0.0990 | 0.4831 | 0.4834 | 0.0172 |
| VEGF | rs3025035 | 0.7935 | 0.4696 | 0.0955 | 0.1905 | 0.3168 | 0.0204 |
| PRL | rs2744117 | 0.0151 | 0.2672 | 0.0048 | 0.2181 | 0.5316 | 0.0206 |
| ESR2 | rs7159462 | 0.2782 | 0.1448 | 0.0222 | 0.9783 | 0.1628 | 0.0248 |
| PRL | rs1156546 | 0.3857 | 0.6563 | 0.0230 | 0.7959 | 0.7049 | 0.0249 |
| SHBG | rs6258 | 0.1838 | 0.3455 | 0.0370 | 0.9560 | 0.3793 | 0.0345 |
| IGFBP1;IGFBP3 | rs13232606 | 0.8012 | 0.3466 | 0.1156 | 0.0788 | 0.1915 | 0.0371 |
| PPARG | rs4135268 | 0.3073 | 0.3787 | 0.0954 | 0.1506 | 0.2094 | 0.0419 |
| ESR2 | rs10137185 | 0.5709 | 0.3182 | 0.0575 | 0.8857 | 0.3399 | 0.0425 |
| PGR | rs569857 | 0.0911 | 0.9383 | 0.0603 | 0.8194 | 0.9058 | 0.0434 |
| PPARG | rs4279078 | 0.9819 | 0.8886 | 0.0190 | 0.8412 | 0.8252 | 0.0442 |
| PPARG | rs2960420 | 0.6411 | 0.3019 | 0.0219 | 0.6607 | 0.2890 | 0.0458 |
| PGR | rs5616 | 0.2156 | 0.9383 | 0.1096 | 0.7671 | 0.2567 | 0.0526 |
| COMT | rs165774 | 0.5625 | 0.3614 | 0.1328 | 0.2836 | 0.5911 | 0.0595 |
| CYP1A1;CYP1A2 | rs2606345 | 0.0442 | 0.0263 | 0.3122 | 0.1386 | 0.0886 | 0.0603 |
| TNF | rs2844482 | 0.2251 | 0.7993 | 0.2360 | 0.2501 | 0.5965 | 0.0645 |
| ESR1 | rs2250122 | 0.0528 | 0.1900 | 0.2557 | 0.0329 | 0.0646 | 0.0688 |
| VEGF | rs699946 | 0.1672 | 0.7300 | 0.0587 | 0.5804 | 0.8725 | 0.0719 |
| SHBG | rs858521 | 0.1140 | 0.6567 | 0.0616 | 0.5377 | 0.5487 | 0.0725 |
| PPARG | rs2920499 | 0.1021 | 0.0684 | 0.1887 | 0.2661 | 0.1545 | 0.0747 |
| PGR | rs481883 | 0.5451 | 0.3780 | 0.3121 | 0.2971 | 0.2977 | 0.0765 |
| COMT | rs5993875 | 0.7110 | 0.1376 | 0.1215 | 0.8547 | 0.1969 | 0.0776 |
| IL6 | rs2069 | 0.9668 | 0.5721 | 0.2069 | 0.4266 | 0.9318 | 0.0816 |
| PGR | rs7106686 | 0.1384 | 0.7675 | 0.0559 | 0.8918 | 0.7610 | 0.0854 |
| PRL | rs9358533 | 0.1048 | 0.3296 | 0.0159 | 0.2445 | 0.5812 | 0.0927 |
| COMT | rs1262 | 0.8547 | 0.0582 | 0.2174 | 0.4017 | 0.0463 | 0.0948 |
| ESR1 | rs3798577 | 0.3489 | 0.2876 | 0.2199 | 0.1425 | 0.5181 | 0.0952 |
| ESR1 | rs1801132 | 0.9799 | 0.8883 | 0.1216 | 0.4633 | 0.7176 | 0.1066 |
| COMT | rs2239393 | 0.4891 | 0.5077 | 0.1740 | 0.7605 | 0.4862 | 0.1085 |
| TNF | rs769177 | 0.0134 | 0.2439 | 0.9702 | 0.0013 | 0.9125 | 0.1160 |
| IGFBP1;IGFBP3 | rs3763497 | 0.5456 | 0.5182 | 0.3000 | 0.4367 | 0.4118 | 0.1211 |
| ESR2 | rs2772163 | 0.5983 | 0.3565 | 0.2189 | 0.7942 | 0.4389 | 0.1280 |
| SULT1A1;SULT1A2 | rs1140 | 0.9282 | 0.6462 | 0.2360 | 0.7818 | 0.3491 | 0.1420 |
| PGR | rs507141 | 0.1860 | 0.2472 | 0.0550 | 0.6771 | 0.2398 | 0.1431 |
| ESR1 | rs3798758 | 0.6162 | 0.6594 | 0.1994 | 0.7568 | 0.7655 | 0.1460 |
| TNF | rs2857708 | 0.5108 | 0.9964 | 0.2603 | 0.4514 | 0.8139 | 0.1464 |
| IGFBP1;IGFBP3 | rs7454 | 0.4945 | 0.1198 | 0.5134 | 0.2392 | 0.2400 | 0.1488 |
| CYP1B1 | rs162550 | 0.9598 | 0.0103 | 0.4358 | 0.1121 | 0.0068 | 0.1500 |
| COMT | rs4680 | 0.9690 | 0.8677 | 0.1956 | 0.8469 | 0.8317 | 0.1601 |
| ESR2 | rs1256114 | 0.3210 | 0.9345 | 0.1400 | 0.7296 | 0.9961 | 0.1628 |
| VEGF | rs1547651 | 0.8222 | 0.1993 | 0.2041 | 0.4245 | 0.2016 | 0.1651 |
| PGR | rs5181 | 0.9633 | 0.6196 | 0.0411 | 0.7517 | 0.5791 | 0.1753 |
| SHBG | rs1642796 | 0.0099 | 0.2834 | 0.7117 | 0.0053 | 0.070 | 0.1754 |
| PRL | rs1205960 | 0.0155 | 0.2348 | 0.0559 | 0.1373 | 0.1362 | 0.1765 |
| VEGF | rs6900017 | 0.8453 | 0.4190 | 0.5653 | 0.1866 | 0.3064 | 0.1766 |
| TGFB1 | rs2241 | 0.2380 | 0.4743 | 0.0883 | 0.4083 | 0.7254 | 0.1778 |
| ESR1 | rs728524 | 0.5870 | 0.7653 | 0.1291 | 0.8756 | 0.7546 | 0.1835 |
| PRLR | rs13354826 | 0.3852 | 0.9873 | 0.5185 | 0.5264 | 0.8763 | 0.1947 |
| PPARG | rs1875 | 0.4261 | 0.1053 | 0.5065 | 0.1899 | 0.9302 | 0.2024 |
| PPARG | rs1181 | 0.5026 | 0.2561 | 0.3389 | 0.3129 | 0.6791 | 0.2072 |
| HSD17B1 | rs597255 | 0.9978 | 0.6500 | 0.1972 | 0.6879 | 0.8103 | 0.2100 |
| CYP1B1 | rs149253 | 0.5128 | 0.9804 | 0.1133 | 0.7790 | 0.9348 | 0.2134 |
| SHBG | rs2543553 | 0.8875 | 0.4961 | 0.2490 | 0.5175 | 0.6472 | 0.2143 |
| COMT | rs2518823 | 0.6942 | 0.5187 | 0.2133 | 0.5592 | 0.4565 | 0.2173 |
| IGFBP1;IGFBP3 | rs2854746 | 0.1982 | 0.3629 | 0.3560 | 0.3460 | 0.5585 | 0.2213 |
| CYP1B1 | rs162557 | 0.9809 | 0.0635 | 0.3194 | 0.3755 | 0.0622 | 0.2221 |
| ESR2 | rs3020443 | 0.8782 | 0.6056 | 0.2391 | 0.8831 | 0.6392 | 0.2221 |
| PGR | rs635984 | 0.4795 | 0.5361 | 0.4587 | 0.6927 | 0.4428 | 0.2266 |
| COMT | rs9306229 | 0.3079 | 0.6858 | 0.0649 | 0.3609 | 0.5463 | 0.2269 |
| PGR | rs474320 | 0.8621 | 0.3375 | 0.2370 | 0.5899 | 0.4279 | 0.2317 |
| IL6 | rs6952 | 0.9335 | 0.2420 | 0.5649 | 0.3380 | 0.4020 | 0.2428 |
| AR | rs5031002 | 0.4702 | 0.1435 | 0.2842 | 0.8389 | 0.2058 | 0.2439 |
| ESR2 | rs1256049 | 0.5297 | 0.2667 | 0.4823 | 0.2595 | 0.4105 | 0.2479 |
| COMT | rs165728 | 0.5313 | 0.5050 | 0.1845 | 0.7561 | 0.5047 | 0.2497 |
| CYP19A1 | rs727479 | 0.1485 | 0.7285 | 0.9604 | 0.0225 | 0.3686 | 0.2510 |
| COMT | rs2020917 | 0.8874 | 0.2497 | 0.6720 | 0.3345 | 0.4518 | 0.2516 |
| PGR | rs5651 | 0.3226 | 0.2472 | 0.1342 | 0.7894 | 0.9900 | 0.2522 |
| TNF | rs9156 | 0.5642 | 0.4019 | 0.2608 | 0.8709 | 0.3950 | 0.2537 |
| ESR2 | rs1152589 | 0.7457 | 0.2832 | 0.3327 | 0.4178 | 0.2311 | 0.255 |
| VEGF | rs833069 | 0.0408 | 0.8841 | 0.0953 | 0.1962 | 0.6280 | 0.2571 |
| HSD17B1 | rs12602084 | 0.4324 | 0.2153 | 0.3583 | 0.3160 | 0.4128 | 0.2676 |
| VEGF | rs3024994 | 0.1046 | 0.5820 | 0.1448 | 0.3279 | 0.4030 | 0.2730 |
| COMT | rs2239395 | 0.5821 | 0.9703 | 0.0971 | 0.9463 | 0.9861 | 0.2741 |
| COMT | rs887200 | 0.7632 | 0.3136 | 0.5046 | 0.6547 | 0.2852 | 0.2761 |
| COMT | rs5993883 | 0.9457 | 0.9984 | 0.4474 | 0.5233 | 0.8563 | 0.2778 |
| PGR | rs477151 | 0.1215 | 0.7209 | 0.3391 | 0.8388 | 0.8604 | 0.2828 |
| ESR2 | rs12435857 | 0.9052 | 0.6777 | 0.4792 | 0.2628 | 0.9032 | 0.2924 |
| ESR2 | rs1571512 | 0.9146 | ̶ | 0.1679 | 0.7323 | ̶ | 0.2971 |
| AR | rs962458 | 0.3607 | 0.2134 | 0.2783 | 0.8771 | 0.2130 | 0.3049 |
| PGR | rs471767 | 0.2202 | 0.3958 | 0.3814 | 0.4697 | 0.4911 | 0.3080 |
| IGFBP1;IGFBP3 | rs903889 | 0.3553 | 0.4971 | 0.3209 | 0.4713 | 0.4620 | 0.3110 |
| COMT | rs933271 | 0.0179 | 0.9388 | 0.9429 | 0.0069 | 0.4356 | 0.3162 |
| IGFBP1;IGFBP3 | rs4619 | 0.1067 | 0.0531 | 0.2585 | 0.8022 | 0.0950 | 0.3214 |
| COMT | rs4333017 | 0.3702 | 0.5337 | 0.9506 | 0.0832 | 0.9297 | 0.3230 |
| PGR | rs11224579 | 0.8557 | 0.4704 | 0.4531 | 0.8862 | 0.5316 | 0.3238 |
| PRL | rs2655417 | 0.3716 | 0.7768 | 0.3083 | 0.5853 | 0.6507 | 0.3286 |
| SHBG | rs2955617 | 0.2959 | 0.7196 | 0.5993 | 0.4934 | 0.6389 | 0.3324 |
| COMT | rs1012157 | 0.1050 | 0.9180 | 0.1890 | 0.4519 | 0.7920 | 0.3366 |
| PGR | rs1824125 | 0.1723 | 0.8176 | 0.1061 | 0.2504 | 0.8911 | 0.3394 |
| COMT | rs1110 | 0.7683 | 0.3835 | 0.6375 | 0.6821 | 0.2431 | 0.3409 |
| CYP1A1;CYP1A2 | rs11072507 | 0.0729 | 0.0281 | 0.7623 | 0.4724 | 0.0578 | 0.3422 |
| ESR2 | rs1887994 | 0.6700 | 0.9187 | 0.8136 | 0.4149 | 0.9260 | 0.3425 |
| SULT1A1;SULT1A2 | rs12445705 | 0.4864 | 0.8110 | 0.4070 | 0.5295 | 0.6607 | 0.3427 |
| PPARG | rs1175543 | 0.8941 | 0.0017 | 0.6255 | 0.4346 | 0.0028 | 0.3563 |
| IL6 | rs10242595 | 0.8570 | 0.8472 | 0.5356 | 0.6154 | 0.8089 | 0.3568 |
| CYP1B1 | rs162556 | 0.6641 | 0.9849 | 0.5270 | 0.5593 | 0.8534 | 0.3572 |
| AR | rs7064188 | 0.9502 | 0.5165 | 0.4522 | 0.6074 | 0.4746 | 0.3579 |
| PPARG | rs2292101 | 0.8071 | 0.8577 | 0.3554 | 0.3358 | 0.9762 | 0.3661 |
| PGR | rs653752 | 0.1578 | 0.2841 | 0.3489 | 0.9716 | 0.4247 | 0.3787 |
| ESR2 | rs1271572 | 0.9305 | 0.6800 | 0.5742 | 0.5397 | 0.5989 | 0.3869 |
| PRL | rs849880 | 0.1798 | 0.7154 | 0.3508 | 0.7721 | 0.6085 | 0.3889 |
| ESR2 | rs1256062 | 0.1568 | 0.4083 | 0.8307 | 0.0908 | 0.7951 | 0.3949 |
| SHBG | rs858518 | 0.2450 | 0.4044 | 0.3078 | 0.9860 | 0.5239 | 0.3970 |
| IGFBP1;IGFBP3 | rs2960436 | 0.2631 | 0.4138 | 0.4655 | 0.9357 | 0.4749 | 0.3983 |
| PRL | rs2655433 | 0.7669 | 0.3074 | 0.9932 | 0.2466 | 0.5094 | 0.4007 |
| PPARG | rs709159 | 0.6453 | 0.4944 | 0.1035 | 0.3070 | 0.7919 | 0.4067 |
| SULT1A1;SULT1A2 | rs4788074 | 0.2119 | 0.8995 | 0.8615 | 0.0336 | 0.5167 | 0.4091 |
| PRL | rs1205961 | 0.6388 | 0.7302 | 0.3334 | 0.7637 | 0.8024 | 0.4140 |
| POU5F1 | rs1447295 | 0.9558 | 0.4431 | 0.6369 | 0.3842 | 0.6706 | 0.4212 |
| IGFBP1;IGFBP3 | rs4724443 | 0.0538 | 0.7340 | 0.7607 | 0.0312 | 0.3924 | 0.4264 |
| CYP1B1 | rs163077 | 0.2190 | 0.1141 | 0.4534 | 0.9812 | 0.1357 | 0.4294 |
| PPARG | rs7963 | 0.5221 | 0.1554 | 0.7550 | 0.3330 | 0.1110 | 0.4329 |
| PGR | rs1046982 | 0.5430 | 0.9128 | 0.2370 | 0.6160 | 0.7826 | 0.4333 |
| IGFBP1;IGFBP3 | rs1995050 | 0.5241 | 0.5154 | 0.4459 | 0.9951 | 0.5860 | 0.4341 |
| CYP1B1 | rs10175338 | 0.2983 | 0.2248 | 0.8357 | 0.4019 | 0.1694 | 0.4377 |
| IGFBP1;IGFBP3 | rs3110697 | 0.2302 | 0.3929 | 0.6865 | 0.1380 | 0.2358 | 0.4381 |
| IGFBP1;IGFBP3 | rs35539615 | 0.0919 | 0.9643 | 0.2890 | 0.4105 | 0.9660 | 0.4381 |
| VEGF | rs25648 | 0.4256 | 0.2636 | 0.2605 | 0.9276 | 0.3792 | 0.4389 |
| VEGF | rs833053 | 0.2132 | 0.7266 | 0.7292 | 0.1298 | 0.9324 | 0.4395 |
| PGR | rs4995 | 0.9939 | 0.3589 | 0.3041 | 0.3049 | 0.3897 | 0.4406 |
| CYP1B1 | rs163078 | 0.2595 | 0.6075 | 0.4578 | 0.7813 | 0.5520 | 0.4411 |
| ESR2 | rs8020646 | 0.6806 | 0.9654 | 0.3672 | 0.8262 | 0.9480 | 0.4413 |
| IGFBP1;IGFBP3 | rs2132570 | 0.3696 | 0.8940 | 0.4784 | 0.5343 | 0.8207 | 0.4469 |
| VEGF | rs879825 | 0.6971 | 0.3873 | 0.8687 | 0.1480 | 0.2466 | 0.4470 |
| PRL | rs6920781 | 0.4745 | 0.5323 | 0.1277 | 0.1846 | 0.4515 | 0.4471 |
| PGR | rs563656 | 0.8342 | 0.4076 | 0.5157 | 0.9361 | 0.4064 | 0.4497 |
| ESR2 | rs7154455 | 0.4919 | 0.5729 | 0.6277 | 0.9921 | 0.6367 | 0.4507 |
| CYP1B1 | rs1800 | 0.2146 | 0.4371 | 0.3145 | 0.9628 | 0.0585 | 0.4529 |
| VEGF | rs3025010 | 0.8349 | 0.2379 | 0.3891 | 0.7697 | 0.2407 | 0.4568 |
| PGR | rs542384 | 0.8850 | 0.1290 | 0.1786 | 0.6995 | 0.1751 | 0.4605 |
| PRL | rs17205146 | 0.5636 | 0.1645 | 0.5318 | 0.6620 | 0.2611 | 0.4642 |
| CYP1A1;CYP1A2 | rs12441817 | 0.8916 | 0.9614 | 0.4701 | 0.9533 | 0.9369 | 0.4684 |
| HSD3B1; HSD3B2 | rs6428830 | 0.2484 | 0.1510 | 0.7541 | 0.4929 | 0.2409 | 0.4742 |
| IL6 | rs10156056 | 0.3847 | 0.8564 | 0.6847 | 0.3798 | 0.7353 | 0.4781 |
| PRL | rs849872 | 0.1085 | 0.3192 | 0.1553 | 0.1459 | 0.2247 | 0.4830 |
| COMT | rs740603 | 0.9943 | 0.3980 | 0.5364 | 0.6914 | 0.4165 | 0.4844 |
| SRD5A2 | rs523349 | 0.3824 | 0.1525 | 0.9284 | 0.1247 | 0.0704 | 0.4894 |
| IGFBP1;IGFBP3 | rs1553009 | 0.1562 | 0.7190 | 0.5211 | 0.6308 | 0.6729 | 0.4937 |
| GHRHR | rs4988 | 0.7603 | 0.4420 | 0.5287 | 0.8091 | 0.2818 | 0.4959 |
| PPARG | rs3856806 | 0.0229 | 0.0840 | 0.1005 | 0.1118 | 0.2697 | 0.4985 |
| CYP1A1;CYP1A2 | rs2470 | 0.5235 | 0.2684 | 0.5146 | 0.6982 | 0.1853 | 0.4996 |
| ESR2 | rs1256044 | 0.9813 | 0.6154 | 0.7063 | 0.7327 | 0.5837 | 0.5000 |
| ESR1 | rs2295190 | 0.2165 | 0.0590 | 0.2211 | 0.2063 | 0.0293 | 0.5004 |
| TGFB1 | rs8110090 | 0.9569 | 0.0866 | 0.4890 | 0.7496 | 0.0912 | 0.5004 |
| PRL | rs6940783 | 0.9601 | 0.0894 | 0.4734 | 0.8925 | 0.1138 | 0.5020 |
| VEGF | rs10434 | 0.4089 | 0.3991 | 0.5260 | 0.8522 | 0.4533 | 0.5037 |
| PGR | rs4734 | 0.9706 | 0.8937 | 0.6709 | 0.7022 | 0.7975 | 0.5058 |
| PRL | rs4711006 | 0.5114 | 0.1453 | 0.7922 | 0.4445 | 0.1452 | 0.5082 |
| VEGF | rs3025 | 0.9356 | 0.5634 | 0.5245 | 0.7652 | 0.5202 | 0.5089 |
| PRL | rs2744105 | 0.2758 | 0.6069 | 0.4950 | 0.8374 | 0.5398 | 0.5094 |
| IL6 | rs6969502 | 0.6702 | 0.1845 | 0.4264 | 0.7920 | 0.1903 | 0.5134 |
| COMT | rs174697 | 0.5524 | 0.4575 | 0.5909 | 0.3591 | 0.3425 | 0.5145 |
| SHBG | rs1619016 | 0.1539 | 0.4365 | 0.7555 | 0.1250 | 0.2534 | 0.5201 |
| PPARG | rs4135247 | 0.9827 | 0.3855 | 0.7399 | 0.6563 | 0.5189 | 0.5212 |
| IGFBP1;IGFBP3 | rs1542818 | 0.7866 | 0.9975 | 0.7420 | 0.6039 | 0.8581 | 0.5225 |
| IGFBP1;IGFBP3 | rs1496497 | 0.8815 | 0.0119 | 0.5554 | 0.4132 | 0.0155 | 0.5259 |
| CYP1B1 | rs1056827 | 0.3832 | 0.2678 | 0.9244 | 0.4906 | 0.2189 | 0.5331 |
| TNF | rs2857605 | 0.1269 | 0.3207 | 0.8881 | 0.2066 | 0.4878 | 0.5368 |
| CYP1B1 | rs1630 | 0.2593 | 0.8221 | 0.4061 | 0.4754 | 0.7774 | 0.5381 |
| PGR | rs555572 | 0.6035 | 0.6181 | 0.7663 | 0.9436 | 0.5647 | 0.5426 |
| ESR2 | rs10144225 | 0.6471 | 0.9364 | 0.8403 | 0.2434 | 0.6231 | 0.5438 |
| TGFB1 | rs10417924 | 0.9993 | 0.9544 | 0.4494 | 0.6448 | 0.9113 | 0.5477 |
| TNF | rs1799964 | 0.7366 | 0.9939 | 0.8441 | 0.8092 | 0.9751 | 0.5485 |
| PGR | rs11224575 | 0.5296 | 0.7433 | 0.5152 | 0.7067 | 0.8335 | 0.5487 |
| IGFBP1;IGFBP3 | rs1065780 | 0.4155 | 0.5168 | 0.6547 | 0.9383 | 0.5085 | 0.5488 |
| CYP1A1;CYP1A2 | rs11631682 | 0.2629 | 0.4035 | 0.8194 | 0.1502 | 0.2514 | 0.5526 |
| TNF | rs13192469 | 0.3144 | 0.1551 | 0.9238 | 0.1690 | 0.3732 | 0.5550 |
| VEGF | rs6899540 | 0.7190 | 0.6630 | 0.5825 | 0.6949 | 0.7007 | 0.5560 |
| COMT | rs887199 | 0.5677 | 0.3705 | 0.9998 | 0.3728 | 0.2946 | 0.5570 |
| VEGF | rs6905288 | 0.1704 | 0.0738 | 0.9711 | 0.4743 | 0.2069 | 0.5605 |
| IGFBP1;IGFBP3 | rs2453836 | 0.2392 | 0.1681 | 0.3942 | 0.6387 | 0.2448 | 0.5628 |
| CYP1A1;CYP1A2 | rs2472297 | 0.5051 | 0.4173 | 0.5774 | 0.7529 | 0.6027 | 0.5736 |
| TGFB1 | rs12983047 | 0.8767 | 0.5792 | 0.8620 | 0.7391 | 0.6504 | 0.5742 |
| PRL | rs2744119 | 0.8366 | 0.5416 | 0.5280 | 0.5760 | 0.5278 | 0.5795 |
| PRL | rs2655418 | 0.3291 | 0.7303 | 0.5841 | 0.6487 | 0.6478 | 0.5816 |
| ESR1 | rs12681 | 0.7084 | 0.1493 | 0.5292 | 0.7769 | 0.2008 | 0.5837 |
| IGFBP1;IGFBP3 | rs6964374 | 0.1453 | 0.2998 | 0.4114 | 0.3909 | 0.4236 | 0.5848 |
| PRL | rs767938 | 0.5602 | 0.6410 | 0.9897 | 0.7458 | 0.8213 | 0.5913 |
| VEGF | rs833058 | 0.1100 | 0.5111 | 0.7073 | 0.8004 | 0.5653 | 0.5923 |
| AR | rs1204038 | 0.3114 | 0.6187 | 0.4448 | 0.7064 | 0.6746 | 0.5927 |
| ESR1 | rs1062577 | 0.1961 | 0.6709 | 0.6546 | 0.7728 | 0.7188 | 0.5933 |
| ESR2 | rs8003490 | 0.2808 | 0.9325 | 0.8519 | 0.1625 | 0.5494 | 0.5944 |
| IGFBP1;IGFBP3 | rs10235181 | 0.9881 | 0.6361 | 0.6525 | 0.5836 | 0.8008 | 0.5983 |
| PRLR | rs37389 | 0.4882 | 0.4029 | 0.8559 | 0.4595 | 0.3035 | 0.6001 |
| CYP17A1 | rs743572 | 0.7650 | 0.4570 | 0.9869 | 0.6491 | 0.4296 | 0.6015 |
| ESR2 | rs1256110 | 0.1394 | 0.7610 | 0.9762 | 0.3663 | 0.5773 | 0.6029 |
| AR | rs5918757 | 0.7707 | 0.4594 | 0.6614 | 0.9619 | 0.4502 | 0.6058 |
| IGFBP1;IGFBP3 | rs6670 | 0.4146 | 0.2357 | 0.7343 | 0.6956 | 0.2802 | 0.6058 |
| SHBG | rs9898876 | 0.9059 | 0.9374 | 0.6993 | 0.5620 | 0.8811 | 0.6067 |
| PGR | rs495997 | 0.9432 | 0.6227 | 0.5830 | 0.9027 | 0.6024 | 0.6127 |
| PRL | rs2655429 | 0.8817 | 0.7275 | 0.5115 | 0.6143 | 0.7585 | 0.6252 |
| ESR1 | rs9479130 | 0.3527 | 0.9267 | 0.3561 | 0.2455 | 0.7621 | 0.6280 |
| PRL | hCV11830642 | 0.2930 | 0.3916 | 0.9206 | 0.2285 | 0.2916 | 0.6408 |
| IL6 | rs11766273 | 0.0701 | 0.1408 | 0.6060 | 0.2205 | 0.3368 | 0.6444 |
| TGFB1 | rs2241 | 0.9965 | 0.9300 | 0.8396 | 0.9027 | 0.8743 | 0.6526 |
| SHBG | rs6259 | 0.5893 | 0.2384 | 0.3603 | 0.5589 | 0.3755 | 0.6554 |
| PRL | rs6929420 | 0.6285 | 0.2742 | 0.4680 | 0.5197 | 0.358 | 0.6632 |
| PGR | rs481775 | 0.1051 | 0.5474 | 0.4732 | 0.7025 | 0.7722 | 0.6665 |
| PPARG | rs13099828 | 0.8532 | 0.2051 | 0.8080 | 0.8783 | 0.2243 | 0.6707 |
| TGFB1 | rs12981053 | 0.7539 | 0.7911 | 0.9885 | 0.7347 | 0.8297 | 0.6716 |
| CYP1B1 | rs162330 | 0.6427 | 0.0968 | 0.5315 | 0.9719 | 0.1253 | 0.6732 |
| CYP1A1;CYP1A2 | rs4886406 | 0.0364 | 0.9948 | 0.8524 | 0.4239 | 0.9178 | 0.6739 |
| IL6 | rs2069833 | 0.3320 | 0.3322 | 0.5453 | 0.8914 | 0.3806 | 0.6760 |
| PRL | rs849876 | 0.1743 | 0.3988 | 0.7566 | 0.6068 | 0.3290 | 0.6834 |
| PPARG | rs17793951 | 0.4229 | 0.2395 | 0.7648 | 0.8280 | 0.2529 | 0.6850 |
| VEGF | rs833052 | 0.4737 | 0.8159 | 0.4038 | 0.4059 | 0.9950 | 0.6866 |
| COMT | rs165849 | 0.4818 | 0.9324 | 0.7797 | 0.8700 | 0.9520 | 0.6880 |
| IGFBP1;IGFBP3 | rs10231774 | 0.4707 | 0.2457 | 0.9470 | 0.3495 | 0.1983 | 0.6927 |
| VEGF | rs998584 | 0.0059 | 0.4056 | 0.3936 | 0.0981 | 0.8125 | 0.6965 |
| CYP1A1;CYP1A2 | rs4886605 | 0.5994 | 0.7045 | 0.7606 | 0.9796 | 0.6372 | 0.6966 |
|  | HCV3289988 | 0.2965 | 0.6909 | 0.8732 | 0.0745 | 0.8706 | 0.7046 |
| ESR2 | rs1256064 | 0.2272 | 0.6076 | 0.9054 | 0.1733 | 0.9680 | 0.7046 |
| IGFBP1;IGFBP3 | rs4146046 | 0.3583 | 0.9323 | 0.6728 | 0.5640 | 0.8718 | 0.7059 |
| TNF | rs928815 | 0.9821 | 0.9064 | 0.8421 | 0.8735 | 0.8760 | 0.7084 |
| ESR2 | rs9440 | 0.8909 | 0.3501 | 0.8004 | 0.9714 | 0.2586 | 0.7095 |
| ESR2 | rs1273196 | 0.5358 | 0.2138 | 0.7685 | 0.5341 | 0.3077 | 0.7106 |
| PPARG | rs1151996 | 0.4551 | 0.0236 | 0.8204 | 0.8514 | 0.0520 | 0.7154 |
| SHBG | rs9913778 | 0.9175 | 0.4413 | 0.5665 | 0.6497 | 0.4138 | 0.7173 |
| CYP1B1 | rs4670813 | 0.8806 | 0.0023 | 0.6104 | 0.9892 | 0.0059 | 0.7202 |
| TGFB1 | rs1146 | 0.9030 | 0.9403 | 0.6060 | 0.5837 | 0.5699 | 0.7218 |
| COMT | rs8141691 | 0.0157 | 0.8793 | 0.7816 | 0.0661 | 0.4941 | 0.7218 |
| HSD3B1; HSD3B2 | rs6428828 | 0.3097 | 0.0650 | 0.8310 | 0.9649 | 0.0954 | 0.7246 |
| IL6 | rs2066 | 0.1825 | 0.0694 | 0.4020 | 0.5943 | 0.4686 | 0.7307 |
| CYP1B1 | rs17490770 | 0.7146 | 0.1783 | 0.7096 | 0.7992 | 0.1686 | 0.7349 |
| IL6 | rs7776857 | 0.0616 | 0.0825 | 0.2389 | 0.1529 | 0.2408 | 0.7359 |
| ESR2 | rs2987983 | 0.5667 | 0.2457 | 0.8478 | 0.7792 | 0.3028 | 0.7365 |
| IL6 | rs1880241 | 0.2798 | 0.4495 | 0.8295 | 0.2445 | 0.2503 | 0.7385 |
| ESR1 | rs9340799 | 0.9716 | 0.2885 | 0.6311 | 0.6938 | 0.4274 | 0.7396 |
| PGR | rs10895068 | 0.7521 | 0.5721 | 0.8273 | 0.5934 | 0.5228 | 0.7400 |
| CYP1A1;CYP1A2 | rs2470890 | 0.1969 | 0.1038 | 0.9584 | 0.8281 | 0.1204 | 0.7434 |
| SHBG | rs1624085 | 0.7382 | 0.5548 | 0.5686 | 0.3717 | 0.8236 | 0.7463 |
| PPARG | rs2920500 | 0.1451 | 0.1570 | 0.7480 | 0.3676 | 0.2840 | 0.7478 |
| PGR | rs619487 | 0.1013 | 0.5609 | 0.5196 | 0.7848 | 0.4762 | 0.7512 |
| TGFB1 | rs8179181 | 0.1798 | 0.0144 | 0.9043 | 0.6943 | 0.0214 | 0.7628 |
| SHBG | rs1799941 | 0.3948 | 0.3992 | 0.9097 | 0.2389 | 0.2623 | 0.7704 |
| FGFR2 | rs2981582 | 0.0306 | 0.5917 | 0.2398 | 0.0170 | 0.2380 | 0.7724 |
| IL6 | rs2069 | 0.4945 | 0.7158 | 0.7562 | 0.5324 | 0.9845 | 0.7763 |
| HSD17B1 | rs676387 | 0.1892 | 0.5816 | 0.9342 | 0.5824 | 0.4222 | 0.7846 |
| PPARG | rs2120825 | 0.1253 | 0.0201 | 0.7662 | 0.2467 | 0.0733 | 0.7865 |
| IL6 | rs6949149 | 0.6240 | 0.0119 | 0.5524 | 0.9551 | 0.0206 | 0.7865 |
| PGR | rs1870 | 0.0666 | 0.7501 | 0.7677 | 0.7087 | 0.5051 | 0.7891 |
| PRL | rs7759000 | 0.6133 | 0.6063 | 0.9950 | 0.7537 | 0.6504 | 0.7893 |
| PPARG | rs4498025 | 0.2980 | 0.8477 | 0.8596 | 0.5684 | 0.9552 | 0.7901 |
| ESR2 | rs1256063 | 0.1626 | 0.0030 | 0.7832 | 0.7786 | 0.0069 | 0.7904 |
| PGR | rs613120 | 0.5745 | 0.2992 | 0.9517 | 0.5031 | 0.2230 | 0.7912 |
| SULT1A1;SULT1A2 | rs4788069 | 0.1774 | 0.8321 | 0.6560 | 0.1676 | 0.8616 | 0.7916 |
| PPARG | rs1899951 | 0.6465 | 0.1708 | 0.8762 | 0.6471 | 0.2308 | 0.8005 |
| PGR | rs3740751 | 0.1726 | 0.4833 | 0.3086 | 0.1307 | 0.8236 | 0.8044 |
| CSHL1 | rs2005172 | 0.8085 | 0.4755 | 0.8234 | 0.5637 | 0.4099 | 0.8089 |
| VEGF | rs833057 | 0.0739 | 0.5677 | 0.5576 | 0.0719 | 0.9569 | 0.8160 |
| SULT1A1;SULT1A2 | rs1968752 | 0.0170 | 0.9788 | 0.2894 | 0.0070 | 0.5006 | 0.8174 |
| TNF | rs2239 | 0.1766 | 0.1016 | 0.6558 | 0.5566 | 0.1515 | 0.8189 |
| IGFBP1;IGFBP3 | rs12702181 | 0.8468 | 0.0729 | 0.9213 | 0.5042 | 0.0543 | 0.8197 |
| IGFBP1;IGFBP3 | rs13223993 | 0.1967 | 0.8939 | 0.6790 | 0.5647 | 0.9821 | 0.8228 |
| ESR2 | rs1256061 | 0.5544 | 0.9638 | 0.9151 | 0.8282 | 0.9600 | 0.8328 |
| AR | rs2361634 | 0.2220 | 0.6644 | 0.4988 | 0.2438 | 0.9343 | 0.8344 |
| IGFBP1;IGFBP3 | rs10241749 | 0.3681 | 0.0375 | 0.7876 | 0.5704 | 0.0657 | 0.8372 |
| ESR2 | rs9444 | 0.5165 | 0.2088 | 0.9336 | 0.8501 | 0.4005 | 0.8424 |
| IGFBP1;IGFBP3 | rs10228265 | 0.6472 | 0.3875 | 0.9649 | 0.6975 | 0.3600 | 0.8425 |
| PPARG | rs13076055 | 0.9089 | 0.6387 | 0.5305 | 0.8740 | 0.6479 | 0.8471 |
| PPARG | rs9833097 | 0.2406 | 0.6109 | 0.9420 | 0.3434 | 0.5113 | 0.8476 |
| CYP1A1;CYP1A2 | rs16972208 | 0.4489 | 0.4927 | 0.8302 | 0.8335 | 0.5883 | 0.8485 |
| ACE | rs4291 | 0.5903 | 0.8333 | 0.9486 | 0.8526 | 0.7813 | 0.8542 |
| PRL | rs849874 | 0.1298 | 0.3192 | 0.3595 | 0.1174 | 0.2137 | 0.8560 |
| IL6 | rs7801617 | 0.8100 | 0.2566 | 0.9303 | 0.7173 | 0.2229 | 0.8570 |
| TGFB1 | rs4803455 | 0.0798 | 0.7786 | 0.9834 | 0.5352 | 0.9330 | 0.8653 |
| PPARG | rs4135 | 0.5619 | 0.6085 | 0.8058 | 0.3644 | 0.6757 | 0.8691 |
| IL6 | rs2056 | 0.9517 | 0.6930 | 0.9268 | 0.2872 | 0.1198 | 0.8707 |
| PPARG | rs13090265 | 0.2075 | 0.7985 | 0.6781 | 0.4634 | 0.7145 | 0.8715 |
| PPARG | rs1152004 | 0.3756 | 0.1781 | 0.7984 | 0.9898 | 0.2087 | 0.873 |
| IL6 | rs1880242 | 0.8595 | 0.2320 | 0.9928 | 0.7406 | 0.3324 | 0.8744 |
| CYP19A1 | rs749292 | 0.3318 | 0.8154 | 0.3248 | 0.0731 | 0.4599 | 0.8767 |
| IL6 | rs17147230 | 0.4597 | 0.5017 | 0.5655 | 0.3746 | 0.7880 | 0.8777 |
| ESR1 | rs2747648 | 0.4508 | 0.1605 | 0.7235 | 0.4083 | 0.2434 | 0.8782 |
| PPARG | rs1152003 | 0.4902 | 0.3754 | 0.5437 | 0.6610 | 0.5005 | 0.8831 |
| PGR | rs492457 | 0.2177 | 0.8989 | 0.7868 | 0.6307 | 0.8789 | 0.8839 |
| CYP1B1 | rs1625 | 0.7414 | 0.0418 | 0.7988 | 0.6363 | 0.0557 | 0.8868 |
| ESR2 | rs17179740 | 0.7844 | 0.5064 | 0.6584 | 0.2522 | 0.3882 | 0.8888 |
| VEGF | rs699947 | 0.1431 | 0.8087 | 0.7685 | 0.8715 | 0.8581 | 0.8915 |
| PPARG | rs17793693 | 0.7124 | 0.4146 | 0.4358 | 0.4136 | 0.6891 | 0.8930 |
| PRL | rs12199382 | 0.8049 | 0.7646 | 0.9800 | 0.4487 | 0.8628 | 0.8946 |
| IL6 | rs12700386 | 0.4699 | 0.7349 | 0.7954 | 0.7770 | 0.7591 | 0.8997 |
| TGFB1 | rs2241713 | 0.2075 | 0.9988 | 0.9223 | 0.7613 | 0.9640 | 0.9008 |
| CYP1A1;CYP1A2 | rs762551 | 0.0760 | 0.4034 | 0.8559 | 0.8088 | 0.4273 | 0.9031 |
| IGFBP1;IGFBP3 | rs2471 | 0.8140 | 0.2419 | 0.8399 | 0.3986 | 0.4177 | 0.9033 |
| TNF | rs2230365 | 0.8271 | 0.5136 | 0.7885 | 0.6670 | 0.5946 | 0.9044 |
| VEGF | rs866236 | 0.3329 | 0.7877 | 0.5653 | 0.1329 | 0.8863 | 0.9150 |
| CYP1B1 | rs1630 | 0.6232 | 0.9715 | 0.4699 | 0.9493 | 0.9767 | 0.9188 |
| PRL | rs849877 | 0.4918 | 0.7753 | 0.4409 | 0.5255 | 0.9225 | 0.9197 |
| VEGF | rs11758547 | 0.0350 | 0.1978 | 0.5536 | 0.0745 | 0.1010 | 0.9235 |
| PPARG | rs4135 | 0.8053 | 0.9485 | 0.8579 | 0.6687 | 0.7289 | 0.9250 |
| CYP1A1;CYP1A2 | rs1048 | 0.0713 | 0.6495 | 0.5744 | 0.4072 | 0.0857 | 0.9265 |
| COMT | rs4646316 | 0.3813 | 0.9803 | 0.8015 | 0.4803 | 0.8813 | 0.9289 |
| HSD3B1; HSD3B2 | rs6686779 | 0.4870 | 0.1151 | 0.8274 | 0.8711 | 0.1545 | 0.9293 |
| PRL | rs849886 | 0.5556 | 0.2465 | 0.7531 | 0.9188 | 0.2312 | 0.9317 |
| IL6 | rs2069861 | 0.1256 | 0.4906 | 0.5951 | 0.3142 | 0.3904 | 0.9332 |
| CYP1B1 | rs1056 | 0.4954 | 0.0734 | 0.5918 | 0.4331 | 0.8617 | 0.9337 |
| PRL | rs9393275 | 0.1003 | 0.0943 | 0.4610 | 0.0988 | 0.2734 | 0.9342 |
| PRL | rs2066265 | 0.7053 | 0.8633 | 0.9304 | 0.9481 | 0.8216 | 0.9344 |
| PGR | rs4754732 | 0.9625 | 0.5262 | 0.6589 | 0.4087 | 0.6234 | 0.9368 |
| ESR2 | rs1256112 | 0.8298 | 0.6261 | 0.8772 | 0.7466 | 0.6090 | 0.9379 |
| SHBG | rs6257 | 0.1798 | 0.8489 | 0.5983 | 0.0776 | 0.5393 | 0.9387 |
| PRL | rs1341239 | 0.8281 | 0.8074 | 0.7520 | 0.9093 | 0.9662 | 0.9443 |
| ESR2 | rs1256033 | 0.5928 | 0.6029 | 0.7991 | 0.6696 | 0.7487 | 0.9458 |
| IL6 | rs4552807 | 0.3776 | 0.6923 | 0.8796 | 0.8662 | 0.7221 | 0.9472 |
| PGR | rs521488 | 0.7380 | 0.8320 | 0.6890 | 0.8666 | 0.9213 | 0.9486 |
| PGR | rs5906 | 0.8418 | 0.5887 | 0.9199 | 0.7377 | 0.6232 | 0.9496 |
| COMT | rs9605 | 0.8317 | 0.8112 | 0.5109 | 0.5255 | 0.9390 | 0.9512 |
| CYP1B1 | rs9341 | 0.5308 | 0.4716 | 0.9695 | 0.7055 | 0.6335 | 0.9519 |
| PRL | rs2655426 | 0.9106 | 0.8560 | 0.8080 | 0.6913 | 0.8139 | 0.9551 |
| PRLR | rs9292573 | 0.1456 | 0.2080 | 0.4344 | 0.2913 | 0.3774 | 0.9559 |
| TNF | rs7769073 | 0.5632 | 0.7483 | 0.7155 | 0.9528 | 0.7849 | 0.9612 |
| AKR1C4 | rs17134592 | 0.3499 | 0.2136 | 0.8598 | 0.8173 | 0.2745 | 0.9634 |
| PPARG | rs4684104 | 0.0874 | 0.4252 | 0.6008 | 0.2445 | 0.6519 | 0.9650 |
| TGFB1 | rs1982072 | 0.1403 | 0.0498 | 0.7979 | 0.8442 | 0.0577 | 0.9661 |
| ESR1 | rs2077647 | 0.2971 | 0.8103 | 0.5560 | 0.0669 | 0.4108 | 0.9663 |
| TNF | rs2256965 | 0.7883 | 0.7938 | 0.7499 | 0.5252 | 0.9706 | 0.9665 |
| COMT | rs4485648 | 0.2598 | 0.6275 | 0.6319 | 0.2482 | 0.8864 | 0.9715 |
| COMT | rs17210001 | 0.6042 | 0.8763 | 0.7410 | 0.6737 | 0.9328 | 0.9747 |
| PPARG | rs17817276 | 0.3940 | 0.1734 | 0.8271 | 0.8929 | 0.1986 | 0.9827 |
| PRL | rs10946546 | 0.6972 | 0.6326 | 0.9059 | 0.4128 | 0.5280 | 0.9894 |
| COMT | rs7289747 | 0.1090 | 0.1851 | 0.6753 | 0.3264 | 0.2691 | 0.9908 |
| PPARG | rs7626560 | 0.9080 | 0.1101 | 0.8244 | 0.7572 | 0.1322 | 0.9929 |
| ADH1C | rs698 | 0.3745 | 0.8816 | 0.6025 | 0.4935 | 0.7030 | 0.9930 |
| PRL | rs12210179 | 0.8428 | 0.5961 | 0.3886 | 0.5086 | 0.8355 | 0.9971 |
| IGFBP1;IGFBP3 | rs9658231 | 0.0699 | 0.2505 | 0.6719 | 0.1270 | 0.1540 | 0.9974 |
| PRL | rs1123886 | 0.7553 | 0.0533 | 0.9358 | 0.8666 | 0.0827 | 0.9993 |
| IGFBP1;IGFBP3 | rs1496499 | 0.5250 | 0.5502 | 0.6810 | 0.5861 | 0.4517 | 0.9994 |
| PPARG | rs1330 | 0.0314 | ̶ | ̶ | 0.2706 | ̶ | ̶ |
| COMT | rs6267 | 0.7413 | ̶ | ̶ | 0.2313 | ̶ | ̶ |
| SULT1E1 | rs11569705 | 0.6474 | 0.5598 | ̶ | 0.7214 | 0.4680 | ̶ |
| SULT1E1 | rs11569712 | ̶ | ̶ | ̶ | ̶ | ̶ | ̶ |
| COMT | rs5992500 | 0.0806 | ̶ | ̶ | 0.4941 | ̶ | ̶ |

**^1^From linear regression models using**  % mammographic density as the outcome variable. Adjusted for age and BMI in a dominant model of inheritance.
